# MitoNGS: an online platform to analyze fish metabarcoding data in high resolution

**DOI:** 10.1093/molbev/msag046

**Published:** 2026-02-19

**Authors:** Tao Zhu, Yukuto Sato, Tsukasa Fukunaga, Masaki Miya, Wataru Iwasaki, Susumu Yoshizawa

**Affiliations:** Department of Integrated Biosciences, Graduate School of Frontier Sciences, The University of Tokyo, Kashiwa, Chiba 277-0882, Japan; Department of Natural Environmental Studies, Graduate School of Frontier Sciences, The University of Tokyo, Kashiwa, Chiba 277-0882, Japan; Faculty of Medicine Research Laboratory Center, University of the Ryukyus, Ginowan, Okinawa 901-2720, Japan; Faculty of Science and Technology, Department of Biosciences and Informatics, Keio University, Yokohama, Kanagawa 223-8522, Japan; Integrated Institute for Regulatory Science, Research Organization for Nano & Life Innovation, Waseda University, Tokyo 162-0041, Japan; Department of Integrated Biosciences, Graduate School of Frontier Sciences, The University of Tokyo, Kashiwa, Chiba 277-0882, Japan; Department of Natural Environmental Studies, Graduate School of Frontier Sciences, The University of Tokyo, Kashiwa, Chiba 277-0882, Japan; Atmosphere and Ocean Research Institute, The University of Tokyo, Kashiwa, Chiba 277-0882, Japan

**Keywords:** metabarcoding, environmental DNA, fish, aquatic biodiversity

## Abstract

Environmental DNA (eDNA) metabarcoding has become a powerful tool for assessing fish biodiversity in aquatic ecosystems. However, accurate species-level identification remains challenging due to incomplete and contaminated reference databases, as well as ambiguous taxa sharing identical barcode sequences. Here, we present MitoNGS, a next-generation platform that succeeds the widely used MiFish pipeline, designed for high-resolution analysis of fish metabarcoding data. MitoNGS addresses these challenges by incorporating more comprehensive references including non-fish species and detailed annotations of heterospecific regions. Additionally, it introduces the “species complex” strategy in conjunction with environmental habitat and geographic occurrence data to resolve ambiguous taxa. Furthermore, MitoNGS expands the functionalities of the legacy MiFish pipeline. It can analyze data from any mitochondrial markers and from Nanopore sequencing platforms. MitoNGS demonstrated excellent performance on our testing datasets from diverse locations, markers, and sequencing platforms. MitoNGS offers a user-friendly, web-based solution for fish detection, biodiversity monitoring, conservation research, and bioresource management. MitoNGS is freely available via https://mitofish.aori.u-tokyo.ac.jp/mito-ngs.

## Introduction

Metabarcoding of environmental DNA (eDNA) targeting mitochondrial genes has emerged as a powerful, efficient, and non-invasive approach for monitoring fish diversity in aquatic environments. The MitoFish database and MiFish pipeline (Iwasaki et al. 2013; Sato et al. 2018; Zhu et al. 2023) were developed to analyze fish eDNA metabarcoding data and have been widely used and cited in recent years (Tsuji et al. 2019; Xiong et al. 2022; Zhu et al. 2023; Yang et al. 2024 ; Jiang et al. 2025 ). However, several challenges remain to enhance the benefits of fish eDNA research, including the application of alternative markers, improved resolution, a multi-sample strategy, and portable sequencing.

The online MiFish pipeline was specifically designed to analyze sequencing data from the MiFish primers (Miya et al. 2015), including MiFish-U and MiFish-E within the 12S rRNA gene for universal fish species and elasmobranchs, respectively. Although these two primers have been proven to be the most efficient and widely used (Xiong et al. 2022), other markers such as Teleo (Valentini et al. 2016) or Riaz (Riaz et al. 2011) are still being utilized in various research. Although a stand-alone version of the MiFish pipeline based on a command-line interface was established two years ago (Zhu et al. 2023), it requires creating and formatting customized reference databases, which is less convenient than the online platform. An updated platform with the capability to handle any mitochondrial markers is essential for expanding fish eDNA research.

Achieving high taxonomic resolution remains a critical challenge in metabarcoding. Species serve as the fundamental unit of biological communities, and ideal metabarcoding outcomes should present solely a comprehensive list of existing species in the samples, which is often impractical. Two primary obstacles hinder species-level identification: incomplete reference databases and ambiguous barcode sequences. Incomplete reference databases, such as those derived from endemic species, lack sequences of invasive and off-target species, resulting in incorrect matching, particularly in instances of low matching identities. The NCBI-nt database (also known as GenBank) (Sayers et al. 2025) includes sequences from all organisms and was directly utilized as a reference database in several fish eDNA research projects (Gao et al. 2025; Plewnia et al. 2025; Rossouw et al. 2025). However, it has been argued to contain errors that could substantially impact the results (Steinegger and Salzberg 2020). On the other hand, ambiguous barcode sequences arise when a single amplicon sequence variant (ASV) matches multiple species with high (99% or even 100%) and identical matching identities. Most algorithms assign the lowest common ancestors (LCAs) to such ASVs (Griffiths et al. 2025; Polanco et al. 2025), which might be overly ambiguous to interpret, as LCAs typically contain numerous other species not belonging to the top hits. The current version of the MiFish pipeline (Sato et al. 2018; Zhu et al. 2023) randomly selects one species, marks it as low confidence, and additionally presents an alternative species. Nevertheless, it remains uncertain whether other alternative species exist. Several studies have assigned a virtual “group” containing solely the top-hit species to the ASV (Rehill et al. 2024; Poiesz et al. 2025). Such groups have also been referred to as “species complex” or “species consensus,” and similar strategies have also been applied in research on amphibians (Lopes et al. 2025) or plants (Ariza et al. 2024). We consider this approach appropriate for reflecting the biological origin of ambiguous ASVs and designate them as “species complexes” in subsequent sections. Although species within the same group have identical barcoding sequences, their environmental habitats such as water depth, salinity, temperature, and geographic distribution may not be identical. Several studies have applied geographic occurrence information to facilitate the filtering of species complexes (Rehill et al. 2024; Anil et al. 2025; Kurniawan et al. 2025; Tibone et al. 2025). The combination of a complete and curated reference database (MitoFish), an off-target database, species complexes, environmental habitat data, and geographic occurrence data will be instrumental in enhancing the taxonomic resolution of metabarcoding.

Incorporating positive controls, negative controls, and biological or technical replicates serves as an effective strategy to mitigate or eliminate the impact of cross-contamination during eDNA metabarcoding. Several analysis pipelines such as VTAM (González et al. 2023) and AMPtk (Palmer et al. 2018) have been developed to address these specialized samples. While the current MiFish pipeline can analyze multiple samples, they are treated uniformly without considering their distinct roles. By incorporating such specialized samples, it will contribute to the reduction of false positive results.

Furthermore, portable Nanopore sequencing devices such as MinION have been successfully applied to on-site eDNA sequencing (Krehenwinkel et al. 2019). This would be a significant advancement in enabling real-time monitoring of fish diversity. Notably, Nanopore sequencing has the capability of sequencing long amplicons, thereby enhancing the distinction between closely related species. However, data analysis of noisy Nanopore reads is challenging and typically requires substantial computational resources. Currently, there are limited eDNA analysis pipelines capable of handling Nanopore reads. The development of an online platform dedicated to analyzing raw Nanopore reads would undoubtedly streamline the entire metabarcoding process. Users would only require a laptop or tablet connected to the Internet, enabling the upload of sequencing results produced by MinION to the online analysis platform for rapid species composition identification.

Here, we have developed MitoNGS, the successor to the MiFish pipeline, to analyze fish eDNA metabarcoding data from any mitochondrial markers and multiple sequencing platforms such as Illumina and Nanopore. MitoNGS utilizes MitoFish and a newly established background database as references to ensure completeness and accuracy. By employing multiple strategies, it generates high-resolution taxonomy annotation results at the species level or species complex level. MitoNGS is publicly available via https://mitofish.aori.u-tokyo.ac.jp/mito-ngs, while the URL of the legacy MiFish pipeline https://mitofish.aori.u-tokyo.ac.jp/mifish will be automatically redirected to MitoNGS.

## Methods

### Reference database

The reference database MitoFish comprises mitochondrial sequences, validated taxonomy labels, environmental habitat, and geographic occurrence data. These data are updated monthly, and historical snapshots are publicly available via https://mitofish.aori.u-tokyo.ac.jp/download/.

#### Sequences and gene annotations

Fish mitochondrial sequences were retrieved from the GenBank database (https://www.ncbi.nlm.nih.gov/nuccore) using the following search strategy: txid7742[Organism:exp] NOT txid32523[Organism:exp] AND mitochondrion[filter] AND ddbj_embl_genbank[filter] NOT uncultured[filter] NOT UNVERIFIED[title] NOT UNVERIFIED_ASMBLY[title] NOT UNVERIFIED_ORG[title] NOT CON[Division]. Downloaded sequences were merged into a single file in FASTA format, with identical sequences combined to reduce redundancy while preserving their accession IDs in the sequence titles. The majority of sequences originate from single mitochondrial genes, and their names were standardized according to Table 1. Sequences containing multiple mitochondrial genes (i.e. complete or partial mitogenomes) and those lacking any gene annotations were reannotated using MitoAnnotator (Iwasaki et al. 2013; Zhu and Iwasaki 2023).

**Table 1:** Uniformed names of mitochondrial genes to facilitate further analysis.

Uniformed names	Description	Alternative names^a^
rRNA genes		
12S rRNA	12S ribosomal RNA	rrnS, rrn12, rns, rrnaS, s-rRNA, SSU, …
16S rRNA	16S ribosomal RNA	rrnL, rrn16, rnl, rrnaL, l-rRNA, LSU, …
Protein-coding genes		
ND1	NADH dehydrogenase subunit 1	NAD1, NADH1, NADH-I, …
ND2	NADH dehydrogenase subunit 2	NAD2, NADH2, NADH-2, …
COXI	Cytochrome c oxidase subunit 1	COX1, COI, CO1, COX-1, …
COXII	Cytochrome c oxidase subunit 2	COX2, COII, CO2, COX-II, …
ATPase8	ATP synthase F0 subunit 8	ATP8, apt8, MTATP8, stp8, ATPase-8, AT8, …
ATPase6	ATP synthase F0 subunit 6	ATP6, MTATP6, ATPase-6, AT6, ATPase-6, …
COXIII	Cytochrome c oxidase subunit 3	COX3, COIII, CO3, COX-III, …
ND3	NADH dehydrogenase subunit 3	NAD3, NADH3, NADH-3, …
ND4L	NADH dehydrogenase subunit 4L	NAD4L, NADH4L, NADH-4L, ND41, NA4L, …
ND4	NADH dehydrogenase subunit 4	NAD4, NADH4, NADH-4, …
ND5	NADH dehydrogenase subunit 5	NAD5, NADH5, NADH-5, NND5, MD5, …
ND6	NADH dehydrogenase subunit 6	NAD6, NADH6, NADH-6, …
Cytb	Cytochrome b	MT-CYB, cyto B, Cyt-B, CYBT, ctb, cob, Cb, …

^a^Names are case-insensitive. For example, there are cases that COXI is written as COi or coi.

#### Taxonomy validation of sequence regions

**Figure 1:**
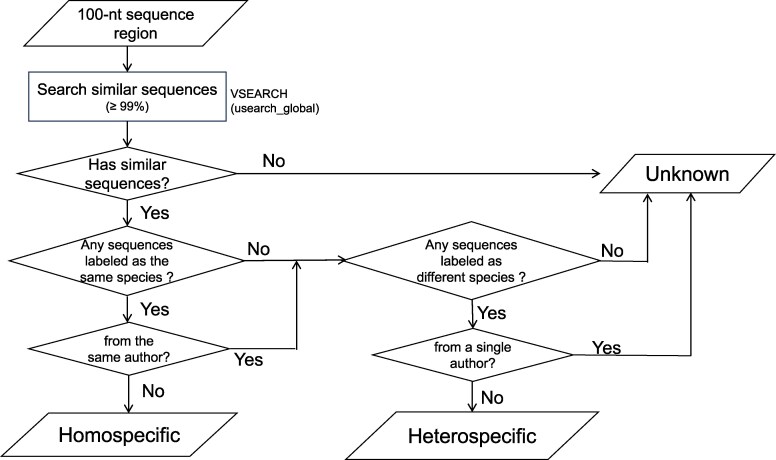
Workflow of judging homospecific and heterospecific mitochondrial gene regions. Heterospecific and unknown regions will undergo validation monthly, with updates to the reference database.

It is crucial to validate the taxonomy labels in reference sequences to prevent false positive metabarcoding outcomes. Recent studies have confirmed the presence of chimeric sequences in GenBank, wherein certain regions within a single sequence originate from heterospecific contamination, while the remaining regions are homospecific and correctly labeled (Sangster and Luksenburg 2020; Claver et al. 2023). From this perspective, mislabeled reference sequences (Li et al. 2018) can be regarded as those containing 100% (or near 100%) heterospecific regions. The primary factor is whether the heterospecific regions overlap with the metabarcoding region, irrespective of the remaining regions. Consequently, we conducted the taxonomy validation on subsequence regions rather than full-length sequences. According to Sangster and Luksenburg (2020)'s research, heterospecific regions in fish mitochondrial genes can be as short as approximately 100 bp. Therefore, we split all the 13 mitochondrial protein-coding genes and two mitochondrial rRNA genes into chunks of 100 bp length. If the final chunk is shorter than 100 bp, it was designated as the final 100 bp of the gene sequence. For each chunk region, taxonomy validation was conducted based on the taxonomy and author information of other homologs with a similarity greater than 99%. Regions with homologs of the same species from different authors were considered homospecific, while regions with only homologs of different species from multiple authors were suggested as heterospecific (Fig. 1).

#### Environmental habitat data

Environmental habitat data for all fish species were sourced from rFishBase (Boettiger et al. 2012). Three primary habitat features, “DemersPelag,” “Salinity,” and “Climate Zone” (Table 2), were selected as they encompass the fundamental aspects of aquatic environments. Notably, these features are available for over 99% of species in rFishBase. Since the species names in the reference database are from the NCBI Taxonomy database, we performed a name mapping process to FishBase via https://www.fishbase.org/tools/upload/CheckName.php.

**Table 2:** Environmental habitat data used in the reference database and MitoNGS.

Type	Values
DemersPelag	Bathydemersal
	Bathypelagic
	Benthopelagic
	Demersal
	Pelagic
	Pelagic-neritic
	Pelagic-oceanic
	Reef-associated
Salinity^a^	Freshwater
	Brackish water
	Saltwater
Climate zone^a^	Subtropical
	Temperate
	Tropical
	Boreal
	Deep-water
	High altitude
	Polar

^a^One species might have multiple values.

#### Occurrence data

Occurrence data with geographic coordinates for all fish species were obtained from Global Biodiversity Information Facility (GBIF, https://www.gbif.org). To minimize redundancy, occurrence data within the same species were deduplicated based on geographic coordinates (latitude and longitude) within a tolerance of 1 degree. Similarly, we conducted a name mapping from the NCBI Taxonomy database to GBIF via https://www.gbif.org/tools/species-lookup.

### Background database

A background database was established to filter metabarcoding sequences originating from off-target amplification of non-fish species or cross-contamination from other metabarcoding experiments. It consists of two parts, (i) nonmitochondrial genes of all species and (ii) mitochondrial genes of non-fish species. The former includes sequences of the 16S rRNA gene of prokaryotes and the 18S rRNA gene of eukaryotes obtained via SILVA v138.2 (Quast et al. 2012) and the internal transcribed spacer (ITS) of eukaryotes obtained via UNITE v10.0 (Abarenkov et al. 2024). The latter part includes all the 13 mitochondrial protein-coding genes and two mitochondrial rRNA genes (12S rRNA and 16S rRNA) obtained via MIDORI2 v264 (Leray et al. 2022), with sequences from fish species excluded by our local scripts. Since the background database serves as a filtering tool rather than a precise identification method for non-fish species, in order to reduce redundancy and speed up the search, sequences were clustered using VSEARCH v2.30.0 (Rognes et al. 2016) at 97% identity, and the centroid of each cluster was kept in the background database.

### Workflow of taxonomy identification

**Figure 2:**
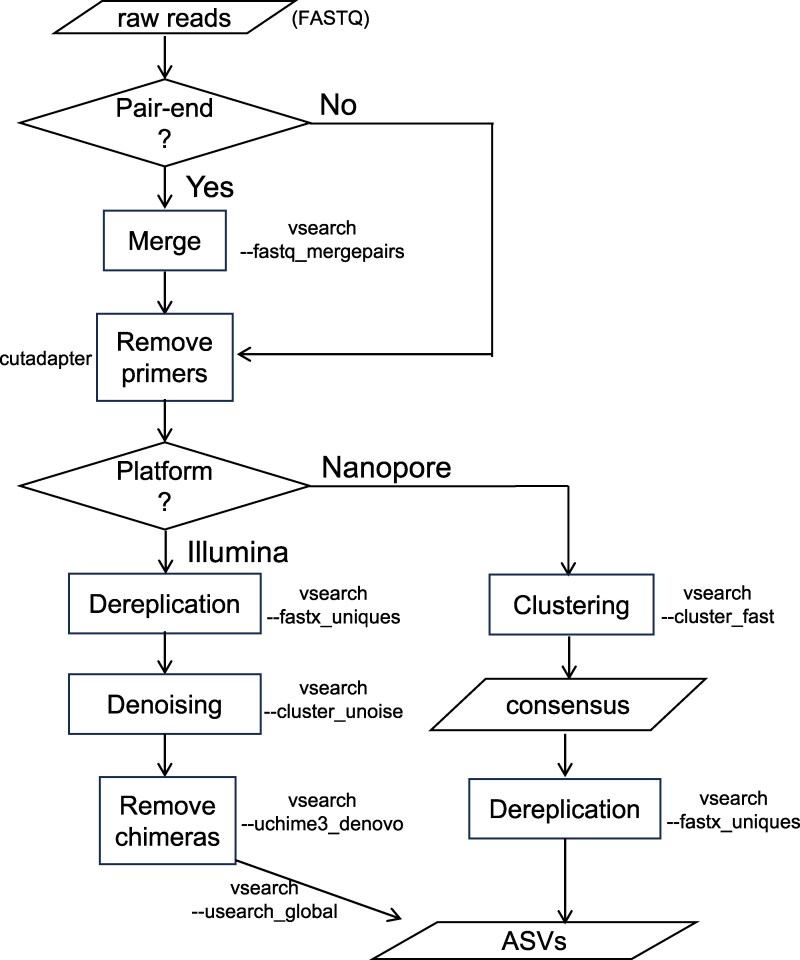
Workflow of generating amplicon sequence variants (ASVs) from raw sequencing reads.

**Figure 3:**
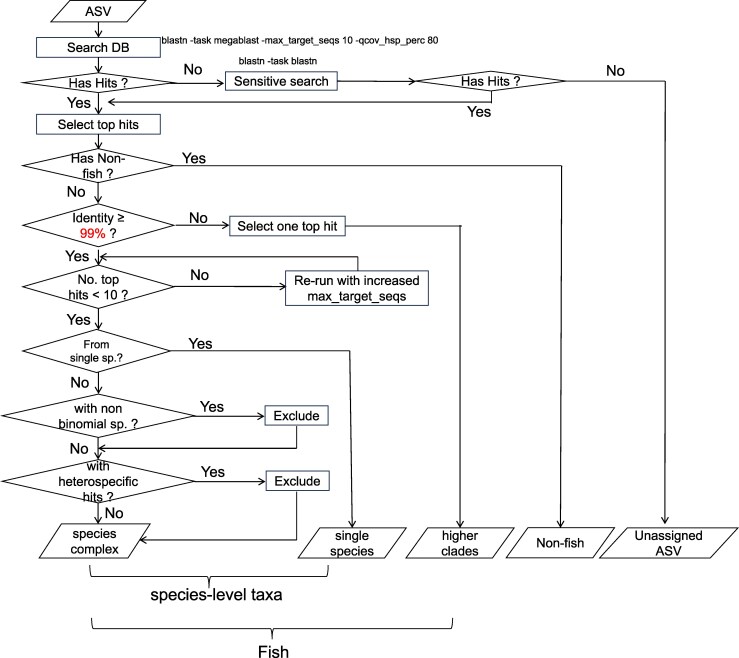
Workflow of taxonomy annotation of amplicon sequence variants (ASVs). 99% is the similarity threshold of annotating ASVs to species level, and this value is adjustable by users. The file containing pseudocode and detailed comments is available via the ASVTaxonomyAnnotation.py file at Zenodo (https://doi.org/10.5281/zenodo.17911248).

The taxonomy identification workflow comprises two distinct phases: the generation of amplicon sequence variants (ASVs) from raw sequencing reads (Fig. 2) and the subsequent taxonomy annotation of ASVs (Fig. 3).

#### ASV generation

The process of generating ASVs from reads of second-generation sequencing platforms, such as Illumina, is similar to the legacy MiFish pipeline (Zhu et al. 2023) except that we replaced USEARCH (Edgar 2016) with the open-source VSEARCH v2.30.0 (Rognes et al. 2016) for denoising, as the latter is more efficient in handling large datasets. For noisy reads generated by third-generation sequencing platforms, such as Nanopore, we utilize the cluster_fast subcommand in VSEARCH to cluster the noisy reads at a 97% identity threshold, employing penalties for gap openings set to “4I/2E” (recommended by VSEARCH developers at https://groups.google.com/g/vsearch-forum/c/qs_9Zo6cc8c/m/jlyFZHNjDwAJ), similar to the default behavior in Minimap2 (Li and Birol 2018). The consensus sequence of each cluster is then used as an ASV for taxonomy annotation.

#### Taxonomy annotation

In MitoNGS, the default similarity threshold for assigning ASVs to the species level is set at 99%, which can be adjusted by users. BLASTN v2.16.0 is employed to search for similar sequences of ASVs in the reference and background databases with parameters “-task megablast -max_target_seqs 10 -max_hsps 1 -qcov_hsp_perc 80.” For each ASV, the top hits with the highest bit score are retained for further analysis. If the number of top hits reaches 10 with identical highest scores and their identities are ≥99%, indicating an unsaturated search, a recursive search method is employed. This method increases the value of “-max_target_seqs” by 50 during each iteration until a hit with a lower score is encountered. For ASVs that do not yield any hits, the parameter “-task megablast” is modified to “-task blastn,” which offers greater sensitivity but incurs a slower processing time, and the search is repeated. These strategies ensure that all ASVs receive their top hits from either the reference or background database in a timely and exhaustive manner.

In the subsequent phase, each ASV was assigned to the species designated in the top hits. For ASVs originating from fish species and exhibiting an identity of ≥99%, if multiple species coexist among the best hits, they undergo a filtering process through two additional steps. Firstly, nonbinomial species, when coexisting with binomial species, are excluded because the former are not yet formally described and named. Secondly, species for which hits located in heterospecific regions were excluded due to insufficient evidence for the labeled taxonomy. If multiple species pass through the filtration process, they are kept as a “species complex” that remains indistinguishable under this metabarcoding marker. Users retain the ability to perform further filtering based on the environmental habitats or geographic occurrences, depending on the research content.

Finally, the taxonomy composition of the entire sample is categorized and presented in three distinct tables: species-level taxa, higher-level taxa, and nonfish taxa. For species-level taxa, abundances are summarized by aggregating the read counts of all ASVs within the same species. For higher-level and nonfish taxa, all ASVs are presented separately, accompanied by the MD5 checksum of the sequence. The pseudocode for taxonomy annotation is available via https://doi.org/10.5281/zenodo.17911248.

#### Controls and replicates

**Figure 4:**
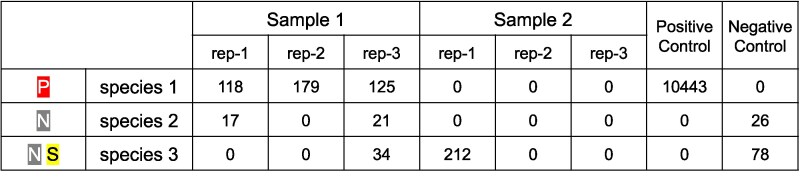
The structure of taxonomic identification results displayed in MitoNGS for multiple samples. Samples (including replicates and controls) are arranged in columns, while species are positioned in rows. The abundance of each species is indicated by the count of reads in the corresponding table cells. The badge “P” signifies the detection of a species in positive controls, “N” denotes its presence in negative controls, and “S” represents its presence in only a single replicate of all samples.

MitoNGS is able to deal with multisample analysis and present results in a table format, where rows represent species (including indistinguishable species complexes) and columns represent samples, including biological/technical replicates, as well as positive/negative controls (Fig. 4). Species detected solely in a single replicate or detected in controls are marked with specific symbols (Fig. 4) to prompt users for manual inspection and filtration. Tables can be exported in CSV format for further analysis.

#### Reanalysis with updated reference

MitoNGS has provided a user-friendly strategy to facilitate reanalysis upon database updates. Unlike the legacy MiFish pipeline, which exclusively accepts raw NGS reads, MitoNGS accepts user-provided ASVs or operational taxonomic units (OTUs) as input data for taxonomy annotation. During the initial analysis, MitoNGS generates annotation results from raw NGS reads and provides users with a unique identifier (job ID) and a downloadable file of ASV sequences. Within 3 months, users can utilize this job ID to retrieve ASVs and parameter settings for reanalysis. After 3 months, old job files are removed from the server, but users can upload ASV sequence files to conduct reanalysis. Consequently, the time-consuming raw read processing and denoising steps are bypassed during the reanalysis process, enhancing overall efficiency.

### Testing datasets

**Figure 5:**
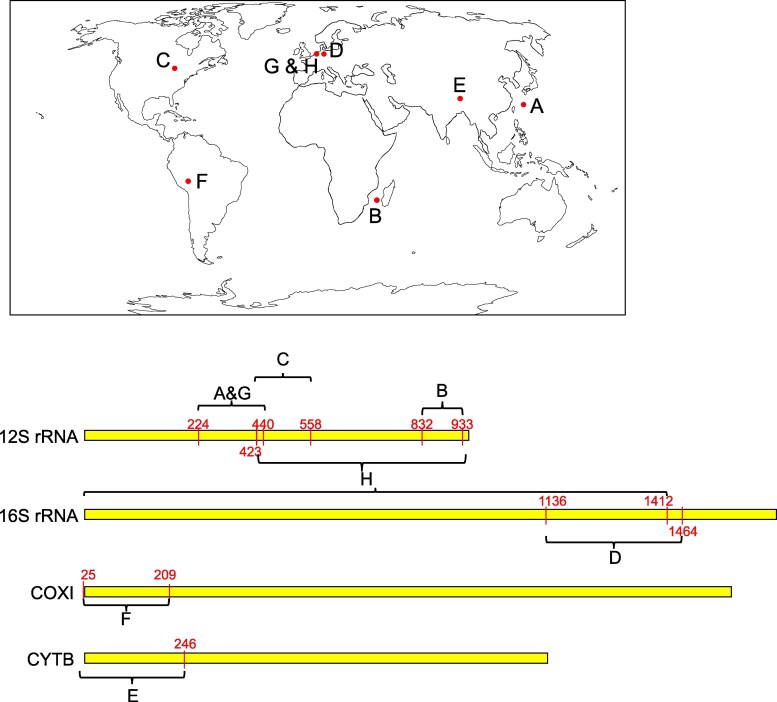
Sampling locations and marker regions of eight testing datasets. Coordinates of marker regions are based on the mitogenome of Japanese eel *Anguilla japonica* (AB038556). (a) Uchidomari river in Okinawa Island, Japan (Sato et al. 2018). Marker is MiFish-U (Miya et al. 2015) in 12S rRNA. (b) Europa Island in the Western Indian Ocean, French Southern and Antarctic Lands (Lamperti et al. 2024). Marker is teleo (Valentini et al. 2016) in 12S rRNA. (c) Boardman Lake in Michigan, USA (Gehri et al. 2021). Marker is Riaz (Riaz et al. 2011; Kelly et al. 2014) in 12S rRNA. (d) Mittelriede and Schunter River in Braunschweig, Germany (Vences et al. 2016). Marker is Vert-16S (Vences et al. 2016) in 16S rRNA. (e) Yarlung Zangbo River in Tibet, China (Feng et al. 2023). Marker is customized by authors using the start region of CYTB. (f) Madre de Dios region of the Peruvian Amazon, Peru (Timana-Mendoza et al. 2025). Marker is Mariac (Mariac et al. 2022) in COXI. (g) North Sea Ray Reef aquarium in Harderwijk, the Netherlands (Doorenspleet et al. 2025). Marker is MiFish-U in 12S rRNA and sequenced by Nanopore. (h) The same samples as G), while marker is customized by authors using a 2 kb region covering 12S and 16S rRNA.

Eight fish eDNA metabarcoding datasets were selected (Fig. 5; Table 3), covering all four widely used genes, 12S rRNA, 16S rRNA, cytochrome c oxidase I (COXI or COI), and cytochrome b (CYTB), and two main sequencing platforms (Illumina and Nanopore). For the dataset of “Europa island in Western Indian Ocean, French Southern and Antarctic Lands” (Lamperti et al. 2024), OTU sequences were readily available and utilized as the initial input so that the result comparison would be easier to conduct in a one-to-one relationship. For other datasets, raw reads were used for annotation.

**Table 3:** Eight datasets for testing the performance of MitoNGS.

Publication	Location	Samples	Target gene	Primer (amplicon length)	Sequencing platform
Sato et al. (2018)	Uchidomari river in Okinawa Island, Japan	Sterivex filter; glassfiber filter	12S rRNA	MiFish-U (∼170)	Illumina
Lamperti et al. (2024)	Europa Island in Western Indian Ocean, French Southern and Antarctic Lands	SPY181529_04; SPY181529_06; CNEG02_09; CNEG202_07	12S rRNA	teleo (∼60)	Illumina
Gehri et al. (2021)	Boardman Lake in Michigan, USA	12S-J1∼3; 12S-JNC1; 12S-neg165∼167; 12S-pos165∼167	12S rRNA	Riaz (∼106)	Illumina
Vences et al. (2016)	Mittelriede and Schunter River, Braunschweig, Germany	Mittelriede river; Schunter river	16S rRNA	Vert_16S (∼260)	Illumina
Feng et al. (2023)	Yarlung Zangbo River, Tibet, China	CBL5; LL5; LZHS; YT9	CYTB	customized (∼260)	Illumina
Timana-Mendoza et al. (2025)	Madre de Dios region of the Peruvian Amazon, Peru	Ronsoco FP2; Shansho FP5; Jimena OL2; Field control, Lab control	COXI	Mariac (∼130)	Illumina
Doorenspleet et al. (2025)	North Sea Ray Reef aquarium, Dolfinarium, Harderwijk, the Netherlands	Dolf1-MiFish; Dolf2-MiFish; Wcontrol-MiFish; PCRcontrol_MiFish_2	12S rRNA	MiFish-U (∼170)	Nanopore
Doorenspleet et al. (2025)	North Sea Ray Reef aquarium, Dolfinarium, Harderwijk, the Netherlands	Dolf1-2kb; Dolf2-2 kb; PCRcontrol_2kb_1; PCRcontrol_2kb_2; Wcontrol-2kb	12S rRNA and 16S rRNA	customized (∼2000)	Nanopore

The taxonomy identification results were compared with those in the original publications. Each species or higher clade reported in the original publication was categorized into one of the following five types:

1. Single (species): relevant ASVs were resolved to species-level by MitoNGS, and the species was consistent with original publication.
2. Multiple (species): relevant ASVs were resolved to species complex level by MitoNGS, and the result was consistent.
3. Clades: ASVs were resolved to higher taxa level by MitoNGS, which was consistent with original publication.
4. Diff: Relevant ASVs were resolved to other species or clades in MitoNGS.
5. Undetected: The reported species or higher clades were not present in MitoNGS.

## Results

### Comprehensive reference database

#### Coverage on species and clades

By June 2025, there were 905,179 sequences in the MitoFish database. Among them, 78.0% were protein-coding and rRNA sequences from 42,996 species, covering all 86 orders, 572 out of all 575 families, and 4,606 out of all 4,772 genera. Scytalinidae (graveldivers), Lacantuniidae, and Hispidoberycidae are the only three families lacking mitochondrial sequences. These families are rarely studied, with fewer than 100 publications to date. Consequently, the reference database (MitoFish) is comprehensive, meeting the needs of global fish eDNA research.

Regarding the four widely used mitochondrial genes—12S rRNA, 16S rRNA, COXI, and CYTB—we observed that while their sequences cover all orders and most families, coverage of genera was lower (Table 4). Notably, for the most widely used 12S rRNA gene, sequences were available in only 77.8% of genera. This is understandable given that studies on 12S rRNA occurred later than those on COXI, which was initially used as a standard marker in many animals (Collins et al. 2019). This gap will be filled by the rapid increase in newly sequenced species in the coming years.

**Table 4:** Sequence coverage of four widely used mitochondrial genes in order, family and genus level

Gene	Orders	Families	Genera
12S rRNA	86 (100%)	548 (95.3%)	3,713 (77.8%)
16S rRNA	86 (100%)	558 (97.0%)	3,909 (81.9%)
COXI	86 (100%)	561 (97.6%)	4,247 (89.0%)
CYTB	86 (100%)	539 (93.7%)	3,600 (75.4%)

By comparing sequences of the above four genes from different fish families, we observed different variation patterns between rRNA genes and protein-coding genes. Conserved regions and hypervariable regions appeared alternately within rRNA genes, but not in protein-coding genes (Text S1), suggesting better metabarcoding performance of rRNA genes (Xu et al. 2024).

#### Heterospecific regions

**Figure 6:**
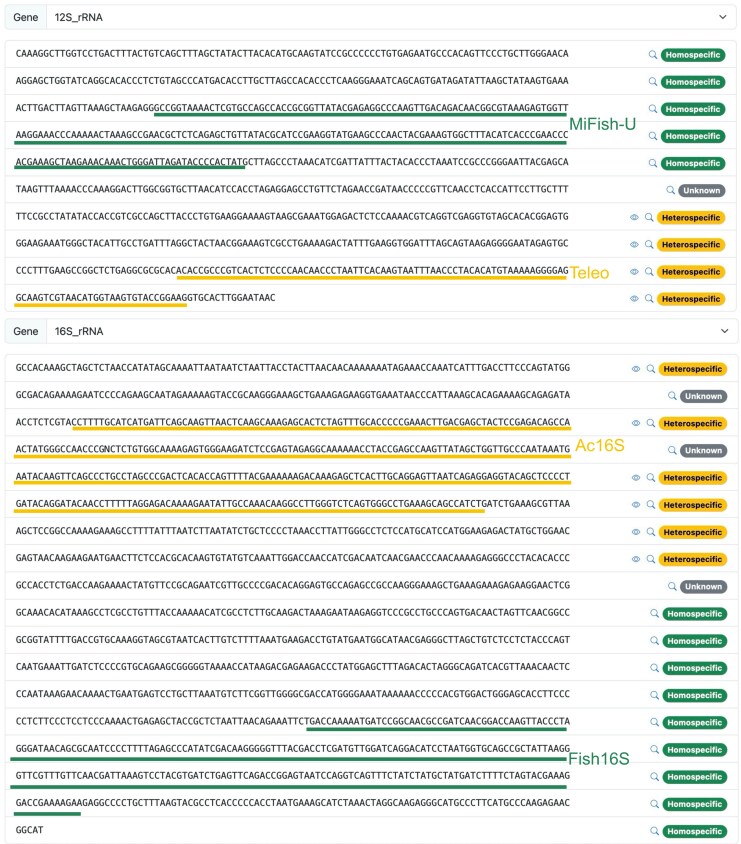
Heterospecific regions of AP006035, the mitogenome of Indo-Pacific/Atlantic sailfish *Istiophorus platypterus*. Amplicon regions of four commonly used metabarcoding primers are displayed: MiFish-U (Miya et al. 2015), Teleo (Valentini et al. 2016), Ac16S (Evans et al. 2016), and Fish16S (Berry et al. 2017).

Among the 706,818 sequences containing protein-coding or rRNA genes, 127,971 contained at least one heterospecific segment. A typical example is one of the full mitogenomes (AP006035) of Indo-Pacific/Atlantic sailfish *Istiophorus platypterus* (Istiophoriformes: Istiophoridae) (Claver et al. 2023). We identified that heterospecific regions occur in the terminal region of 12S rRNA gene and the initial region of the 16S rRNA gene with ≥99% similarity to multiple *Engraulis* species (Clupeiformes: Engraulidae). Metabarcoding markers located at the initial region of 12S rRNA or the terminal region of 16S rRNA would function normally, while those located at the terminal region of 12S rRNA or the initial region of 16S rRNA would be affected (Fig. 6).

#### Environmental habitat and geographic occurrence data

Environmental habitat data and geographic occurrence data were retrieved from FishBase and GBIF, respectively, which primarily contain binomial species. Among the 42,996 species with protein-coding or rRNA sequences in the reference database, there were 22,810 binomial species, among which 22,075 (96.8%) are available with environmental habitat data from FishBase and 21,247 (93.1%) with occurrence data from GBIF. As expected, nonbinomial species seldom have environmental habitat data (0.17%) since they are not included in FishBase. Conversely, the proportion of nonbinomial species with occurrence data was higher (15.9%) because GBIF also includes occurrence records based on DNA evidence.

### Improved metabarcoding results in species-level resolution

#### The benefit of comprehensive reference database

**Figure 7:**
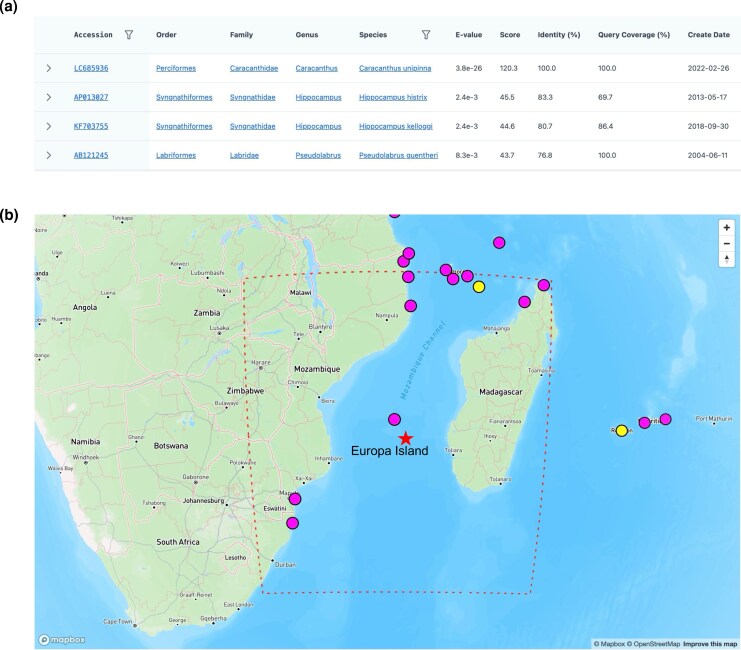
Example of a higher clade (superclass: Actinopterygii) reannotated at species-level (*C. unipinna*). The 66 bp OTU (CCCCGATACATCACCACTCGTATATTAACAAGCCTAGATAAAATACTGAGGGGAGGCAAGTCGTAA) was from the dataset of Europa Island in Western Indian Ocean (Lamperti et al. 2024). a). BLAST results (screenshot via MitoFish at https://mitofish.aori.u-tokyo.ac.jp/blast/simple) of the OTU to top four hits. Similarities of all other three hits are lower than 85%. b) The position of the sampling site Europa Island (marked with a star) and the occurrence records of *C. unipinna* (screenshot via MitoFish at https://mitofish.aori.u-tokyo.ac.jp/detail/species/1146959). Pink points indicate observations of specimen materials (such as those used for sequencing) while yellow points indicate observations of live organisms.

MitoNGS yields consistent results across all eight testing datasets (Table 5). Notably, 32 cases were identified where ASVs or OTUs initially annotated at higher taxonomic levels were subsequently reannotated to the species level (Tables S1c, S2d, S3c, S4c, S7c, and S8c). One notable case is a 66bp-OTU sequence from the dataset of Europa Island in the Western Indian Ocean (Lamperti et al. 2024). Initially annotated as the superclass “Actinopterygii” (Table S2d), the sequence underwent reannotation to the species *Caracanthus unipinna* (Perciformes: Caracanthidae) using MitoNGS. The reference sequence (LC685936) of the best match was published in February 2022, later than the reference database version (March 2020) used in the original publication. It was the only one with high similarity to the OTU sequence, and the species *C. unipinna* has occurrence records near the sampling location (Fig. 7), corroborating the accuracy of the reannotation. For cases where species (or higher taxa) were different from the original results, the majority of them were also improved due to higher matching similarity in the updated references (Tables S1d, S2e, S3e, S7e, and S8d). This demonstrates the significant utility of employing the most recent and comprehensive reference database in achieving high-resolution taxonomy identification outcomes.

**Table 5:** Comparison of taxonomic identification results of MitoNGS and the original literatures.

Location (primer)	No. of taxa in origindal publication	Relevant taxa by MitoNGS^a^				
		Consistent			Diff.	Undetected
		Single sp.	Multiple sp.	Clades		
Uchidomari river in Okinawa Island, Japan (MiFish-U-12S)	26 (species); 1 (clade)	18 (species)	5 (species); 1 (clade)	0	3 (species)	0
Europa Island in Western Indian Ocean, French Southern and Antarctic Lands (teleo-12S)	56 (species); 23 (clades)	40 (species); 9 (clades)	5 (species); 4 (clades)	1 (species); 9 (clades)	10 (species); 1 (clade)	0
Boardman Lake in Michigan, USA (Riaz-12S)	13 (species); 6 (clades)	8 (species); 1 (clade)	4 (species); 4 (clade)	0	1 (species) 1 (clade)	0
Mittelriede and Schunter River, Braunschweig, Germany (Vert-16S)	14 (species)	10 (species)	2 (species)	1 (species)	0	1 (species)
Yarlung Zangbo River, Tibet, China (customized-CYTB)	27 (species)	21 (species)	5 (species)	0	0	1 (species)
Madre de Dios region of the Peruvian Amazon, Peru (Mariac-COXI)	25 (species); 19 (clades)	19 (species); 5 (clades)	4 (species); 3 (clades);	10 (clades)	2 (species); 1 (clade)	0
North Sea Ray Reef aquarium, Dolfinarium, Harderwijk, the Netherlands (MiFish-U-12S)	15 (species); 2 (clades)	12 (species); 1 (clade);	2 (species); 1 (clade)	0	1 (species);	0
North Sea Ray Reef aquarium, Dolfinarium, Harderwijk, the Netherlands (customized-12S-16S)	19 (species)	18 (species)	0	0	0	1 (species)

See Table S1–S9 for the full lists of taxa.

^a^The definition of these five categories was explained in the methods section.

#### Species complexes and further filtration using heterospecific information

**Figure 8:**
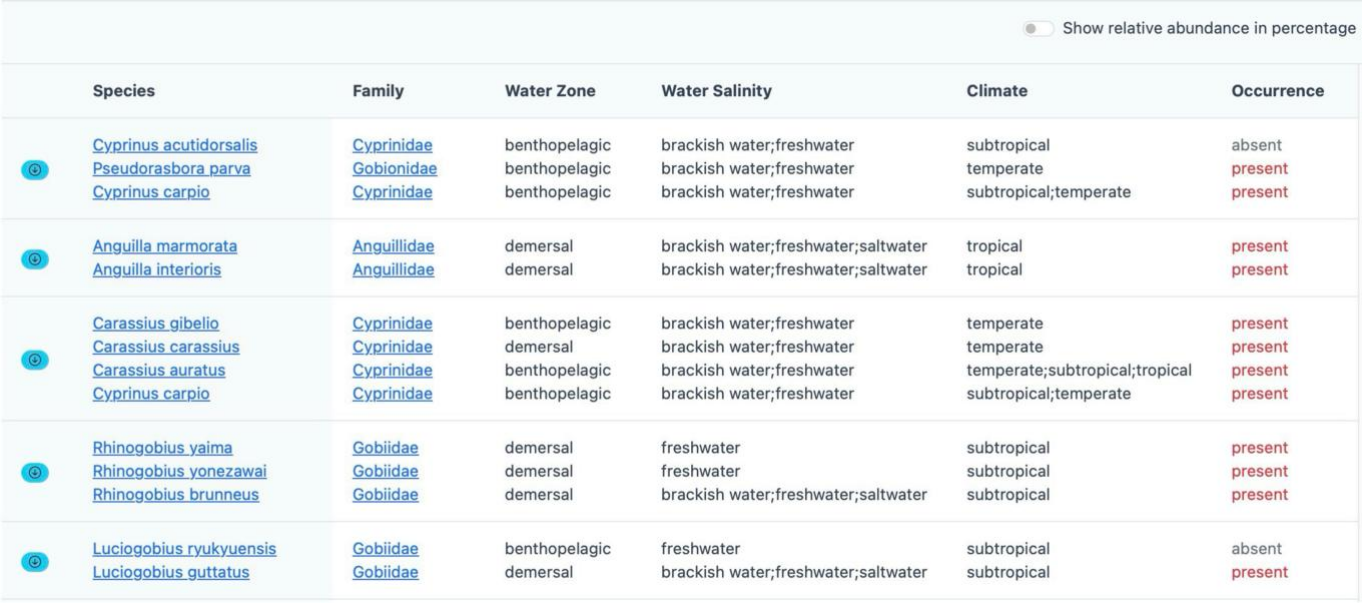
Species complexes detected in MitoNGS. This is a screenshot of the analysis results of the dataset of Uchidomari river in Okinawa Island, Japan (Sato et al. 2018). Species sharing identical ASVs are displayed in the same row, with environmental habitat and occurrence data shown afterward. The attached download button in the leftmost column enables users to download all the mitochondrial sequences of these species to conduct further analysis, such as designing species-specific qPCR primers.

Except for the only dataset with long amplicons (2 kb), all the remaining seven datasets include species complexes where multiple species share identical metabarcoding regions (Table 5). This is reasonable, as short markers inherently exhibit reduced discriminability. In the result table, environmental habitat data and geographic occurrence data were presented to facilitate further filtering, and all related reference sequences of these species could be downloaded (Fig. 8).

As indicated in Fig. 3, heterospecific information is employed to facilitate the filtering of species complexes. We compared the results with those that did not apply this filtering and indeed identified 10 cases where unambiguous assignments were obtained by heterospecific filtration (Table S9). In these cases, ASVs had top hits from multiple species, among which only one species is verified to be nonheterospecific, demonstrating that the inclusion of heterospecific information does indeed contribute to the enhancement of taxonomy resolution.

### Discovery of novel fish species and off-target amplifications

Fifteen novel fish species were detected in three datasets (Table 6, Tables S1e, S6c, S7e, and S9b). Among these, 10 species have occurrence records near the sampling regions, indicating true positive discoveries. The remaining five species lacking occurrence evidence might be invasive species.

**Table 6:** Novel fish species and off-target products detected by MitoNGS.

Location (primer)	No. of novel fish	No. of nonfish species
Uchidomari river in Okinawa Island, Japan (MiFish-U-12S)	1	1 (turtle^a^)
Europa Island in Western Indian Ocean, French Southern and Antarctic Lands (teleo-12S)	0	2 (human^a^, sea urchins^a^)
Boardman Lake in Michigan, USA (Riaz-12S)	0	4 (human, birds, rodents)
Mittelriede and Schunter River, Braunschweig, Germany (Vert-16S)	0	3 (frogs, boar)
Yarlung Zangbo River, Tibet, China (customized-CYTB)	6	1 (human)
Madre de Dios region of the Peruvian Amazon, Peru (Mariac-COXI)	0	4 (tapir^a^, boar, crustacean, frogs^a^)
North Sea Ray Reef aquarium, Dolfinarium, Harderwijk, the Netherlands (MiFish-U-12S)	7	0
North Sea Ray Reef aquarium, Dolfinarium, Harderwijk, the Netherlands (customized-12S-16S)	1	0

Only species satisfying all the following criteria were counted. (1) unambiguous; (2) identities higher than 99%; (3) not detected in negative controls; (4) detected in at least two replicates if available; (5) relevant BLAST hits not located in heterospecific regions (for fish species).

^a^Reported in original publications

Non-fish species with identity ≥99% were detected in six datasets (Table 6, Tables S1a, S2a and e, S3f, S4d, S5d, and S6f), encompassing a range of taxa such as invertebrates, amphibians, and mammals. In fact, nonfish taxonomies were also detected in the remaining two datasets, albeit with a lower identity or only present in a single replicate within samples, potentially indicating contamination. Unassigned ASVs (Fig. 3) are absent from all eight datasets.

## Discussion

### Excellent performance

In all testing datasets, taxonomic identifications are consistent with the original ones, while most inconsistent cases were actually improved annotations due to our more comprehensive reference database. Notably, the analysis speed is fast, particularly for Nanopore reads. Typically, generating consensus sequences from raw Nanopore reads is time-consuming. In our testing datasets, the Dolf2-2 kb library from North Sea Ray Reef aquarium in the Netherlands (Table 3) contains 141,302 long reads with an average length of 2 kb. MitoNGS completed the entire analysis using 30 threads on our server within 8 min. In contrast, the DECONA pipeline used in the original publication (Doorenspleet et al. 2025) required over 2 h to run using the same number of threads on the same server. For regular Illumina datasets, MitoNGS is also more efficient than the legacy MiFish pipeline (Zhu et al. 2023) or other common workflows such as the DADA2/DECIPHER pipeline (Callahan et al. 2016; Murali et al. 2018). The execution time measured based on the MiFish testing dataset (Sato et al. 2018) for MitoNGS, legacy MiFish, and DADA2/DECIPHER was 4, 7, and 25 s, respectively. In summary, MitoNGS achieves a favorable cost–performance ratio by balancing the speed and accuracy.

Although there were five undetected cases (Table 6), all of them were plausible due to insurmountable obstacles. *Cyprinus carpio* in the dataset of Mittelriede River, Germany (Table S4a), was at low abundance (9). Although the abundance of *Triplophysa stolickai* in the dataset of Yarlung Zangbo River, Tibet, China (Table S5a) was higher (66 in sample LZHS), we failed to detect any sequences with high similarity (≥98% based on the authors’ methods) with *Triplophysa stolickai*. Regarding *Chelidonichthys lucerne* in the Nanopore 2 kb libraries (Table S8a), we found that *Chelidonichthys lucerne* lacked long reference sequences by June 2025. The longest rRNA gene, ON000326, was 1,126 bp, resulting in only ∼50% query coverage of the ∼2 kb Nanopore reads, and was therefore not included in the final results. Except for the dataset of Europa Island in French Southern Lands (Lamperti et al. 2024), ASV (or OTU) sequences were not publicly available for the remaining seven datasets, making the one-to-one comparisons difficult. It would be beneficial for all metabarcoding-related research to make amplicon sequences publicly available, as they are computationally intensive but reference-independent. This would facilitate downstream validations, particularly on updated references.

### Reference gaps

Fish ASVs with similarities below 99% were detected in all eight datasets, suggesting the presence of potentially unknown fish species not included in the reference database. To further investigate, in each dataset, we manually inspected five ASVs with the lowest similarities by searching them in GenBank. The results indicated that for COXI metabarcoding, ASVs with extremely low similarities (<80%) might originate from bacterial COXI genes (similarities of ∼90%). Given the limited data resources for accurate bacterial COXI genes, they were not included in the background database. This omission presents an opportunity for future efforts in MitoFish and MitoNGS. For all other genes, no novel hits were found in GenBank, suggesting that plenty of fish species are yet to be discovered and/or sequenced.

### Strategies to improve taxonomy resolution

For ASVs with identical top hits from multiple species, MitoNGS displays species complex instead of LCA. Among all the species complexes detected in our testing datasets, except for genus *Gomphosus* (Table S2b) and *Pomoxis* (Table S5b), which each contains only two species, all other clades include additional species that do not belong to the species complex. In addition, by comparing the results of MitoNGS and the DADA2/DECIPHER pipeline (Callahan et al. 2016; Murali et al. 2018) using the testing dataset of Uchidomari river, Okinawa, Japan (Sato et al. 2018), we found that in most cases, all the sequences from the annotated species complex formed a monophyletic clade and were divergent from other species belonging to the same genus or family (Text S2). Each species complex could be regarded as a micro-LCA that is different from traditional taxonomy ranks such as genus or family. They definitely provide more detailed information on the true biological source of ASVs.

Furthermore, MitoNGS employs binomial status and heterospecific information to filter species complexes (Fig. 3). Additionally, environmental habitat and geographic occurrence data within groups are also displayed (Fig. 8). MitoNGS does not automatically exclude species lacking occurrence records near sampling locations, as occurrence data are often incomplete. For instance, in the testing dataset of Uchidomari river, Okinawa, Japan (Table S1c), *Luciogobius guttatus/ryukyuensis* were detected. Although the latter lacked occurrence records with geographic coordinates, there were 91 records in GBIF with the area roughly labeled as “Japan” by the end of June 2025, suggesting that *Luciogobius ryukyuensis* could still be the correct identification. Such incompleteness might be more significant for species in undersampled regions such as deep sea or tropical rivers. There are no universally accepted guidelines for whether to retain or exclude species with varying habits or occurrence patterns, and this decision should depend on the research objectives and prior knowledge of sampling environments. We hope such bias will be resolved with the accumulation of updated occurrence data from various data sources and community feedback.

We also observed that certain species within some groups could form interspecific hybrids (Tables S1b, S1d, S2d, S3b, S3c and S5b), indicating insufficient reproductive barriers. From another perspective, such species complexes could be considered as “virtual species.” Currently, data on interspecific hybrids and their evidence levels are scarce. In the future, with the accumulation of hybrid cases, species based on genuine reproductive barriers could be constructed to serve as the basic unit of metabarcoding results.

It should be noted that the “species complex” strategy proposed in MitoNGS is a compromise for short markers. Although further filtering could be conducted via environmental habitat or geographic data, there are still many cases where distinct species share identical ecological niches. Long-read-based metabarcoding demonstrated exceptional performance in resolving closely related species. In our testing dataset, a 2 kb Nanopore library successfully identified all the species unambiguously (Table S8). The primary challenge lies in the scarcity of long reference sequences, which will be addressed with the accelerated accumulation of complete mitogenomes.

## Conclusion

MitoNGS is an integrated, web-based platform for the analysis of fish metabarcoding data across diverse mitochondrial loci. By combining curated reference databases, heterospecific region annotation, off-target filtering, environmental metadata, and species occurrence information, MitoNGS enables high-resolution, species-level taxonomic assignments. As the successor to the MiFish pipeline, it addresses several long-standing challenges in eDNA analysis, including ambiguous barcodes and limited marker compatibility. With its flexible input formats and support for long-read sequencing technologies, MitoNGS is poised to advance the field of aquatic biodiversity monitoring and support a wide range of ecological, conservation, and applied research efforts.

## Supplementary Material

msag046_Supplementary_Data

## Data Availability

Data are available at the Supplementary materials, Zenodo (https://doi.org/10.5281/zenodo.17911248), and our server (https://mitofish.aori.u-tokyo.ac.jp).
