# Supplementary material for: MitoNGS: an online platform to analyze fish metabarcoding data in high resolution
Source: Mol Biol Evol. 2026 Feb 19;43(3):msag046. doi: 10.1093/molbev/msag046 (PMC12961187; doi:10.1093/molbev/msag046)
Supplement: msag046_Supplementary_Data [file msag046_supplementary_data.zip › mitoNGS-20260106 Text S1.docx]

**MitoNGS: an online platform to analyze fish metabarcoding data in high-resolution**

Supplementary Text S1. Variation patterns on different mitochondrial markers.

**Methods**

For each of the four commonly employed mitochondrial genes - 12S rRNA, 16S rRNA, cytochrome c oxidase I (COI or COXI), and cytochrome b (CYTB) - a single representative sequence was chosen for each family of ray-finned fishes (Actinopterygii, comprising over 95% of extant fish species) based on the following criteria:

1) the sequence contained the fewest heterospecific regions;

2) when multiple sequences met the first criterion, the longest sequence was selected.

Multiple sequence alignments for each gene were performed using MAFFT v7.505 (Katoh and Standley 2013). Mean pairwise sequence identities were computed using sliding windows of 20 bp with a step size of 5 bp, treating gaps as mismatches. Conserved and highly variable regions were visualized as scatter plots derived from identity values across each gene.

The binding positions of widely used metabarcoding primers were mapped onto the gene regions using the Japanese eel (*Anguilla japonica*) sequence (GenBank accession AB038556) as a reference. The choice of reference sequence did not affect the results.

| **Primer Name** | **Amplicon Region on Reference** | **Sequences (5’ -> 3’)** | **Literature** |
| --- | --- | --- | --- |
| 12S rRNA | | | |
| MiFish-U | 245~413 | F: GTCGGTAAAACTCGTGCCAGC  R: CATAGTGGGGTATCTAATCCCAGTTTG | (Miya, et al. 2015) |
| 12S-V5 (Kelly-12S) | 435~540 | F: ACTGGGATTAGATACCCC  R: TAGAACAGGCTCCTCTAG | (Kelly, et al. 2014) |
| AcMDB07 | 621~901 | F: GCCTATATACCGCCGTCG  R: GTACACTTACCATGTTACGACTT | (Bylemans, et al. 2018) |
| Teleo | 849~910 | F: ACACCGCCCGTCACTCT  R: CTTCCGGTACACTTACCATG | (Valentini, et al. 2016) |
|  | | | |
| 16S rRNA | | | |
| Ac16S | 228~564 | F: CCTTTTGCATCATGATTTAGC  R: CAGGTGGCTGCTTTTAGGC | (Evans, et al. 2016) |
| Vert16S | 1161~1443 | F: AGACGAGAAGACCCYDTGGAGCTT  R: GATCCAACATCGAGGTCGTAA | (Vences, et al. 2016) |
| Fish16S (Shaw) | 1164~1247 | F: CGAGAAGACCCTWTGGAGCTTIAG  R: GGTCGCCCCAACCRAAG | (Shaw, et al. 2016) |
| Berry-fish | 1166~1386 | F: GACCCTATGGAGCTTTAGAC  R: CGCTGTTATCCCTADRGTAACT | (Berry, et al. 2017) |
|  | | | |
| COXI | | | |
| Leray | 393~705 | F: GGWACWRGWTGRACWITITAYCCYCC  R: TAIACYTCIGGRTGICCRAARAAYCA | (Wangensteen, et al. 2018) |
|  | | | |
| Cytb | | | |
| Minamoto | 168~402 | F: TTCCTAGCCATACAYTAYAC  R: GGTGGCKCCTCAGAAGGACATTTGKCCYCA | (Minamoto, et al. 2012) |

**Results**

Within the 12S rRNA (Figure S1) and 16S rRNA (Figure S2) genes, conserved regions (sequence identity >95%) alternate with hypervariable regions (sequence identity <65%) along the gene region.


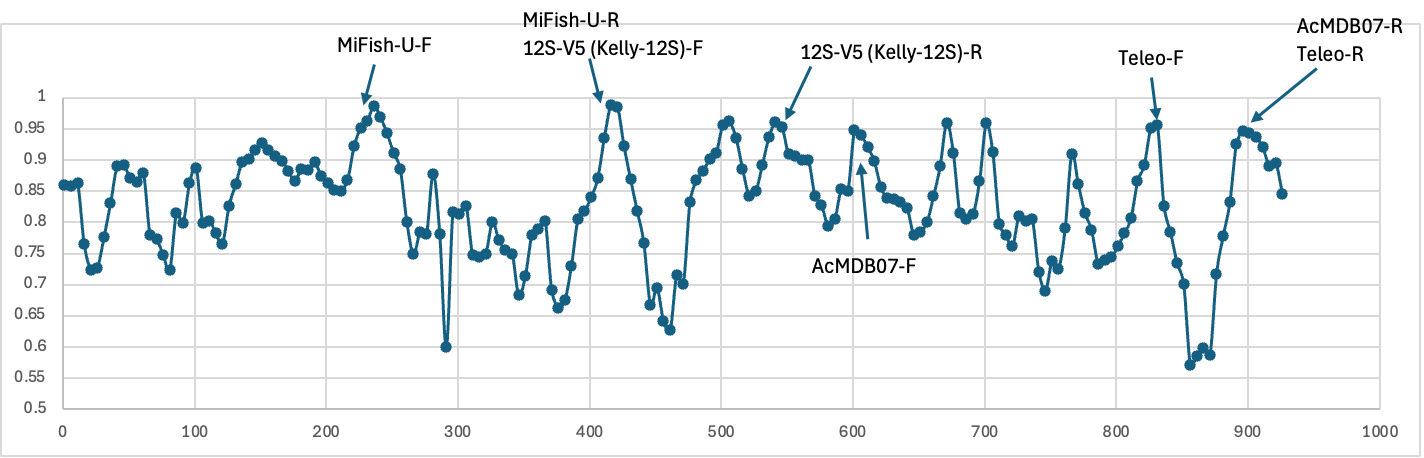


Figure S1. Mean identities along the 12S rRNA gene of 433 families of ray-finned fishes.


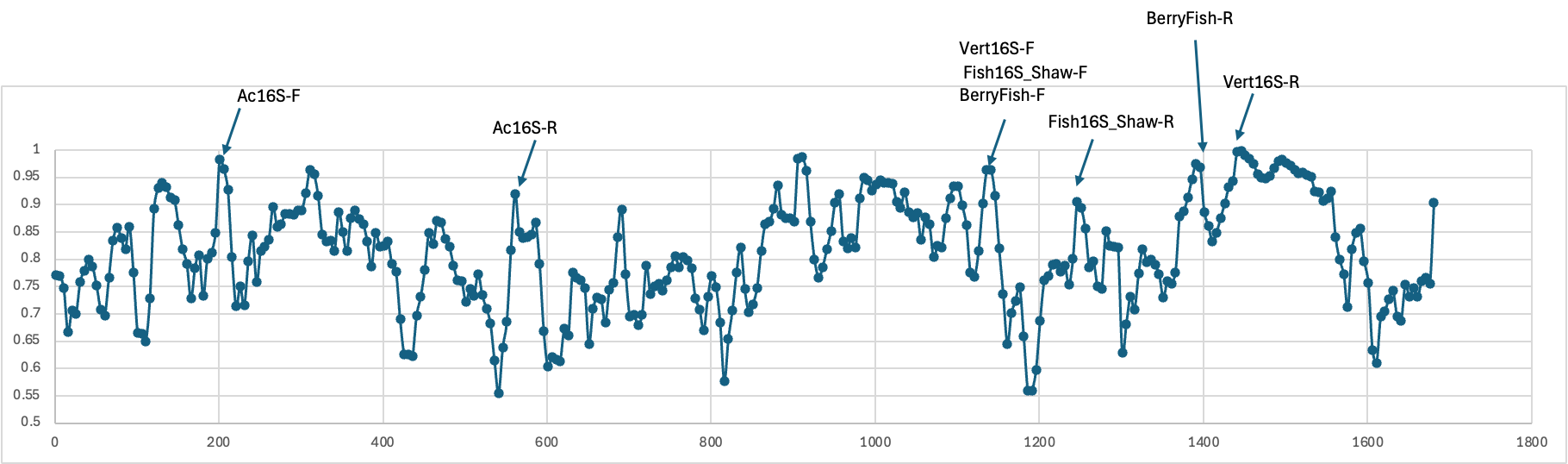


Figure S2. Mean identities along the 16S rRNA gene of 418 families of ray-finned fishes.

In contrast, conserved regions were rarely detected in the COXI (Figure S3) and Cytb (Figure S4) genes, indicating that the use of degenerate primers is required for these loci. Moreover, COXI exhibited a general lack of pronounced hypervariable regions across its entire length, which likely contributes to its reduced effectiveness in fish metabarcoding.


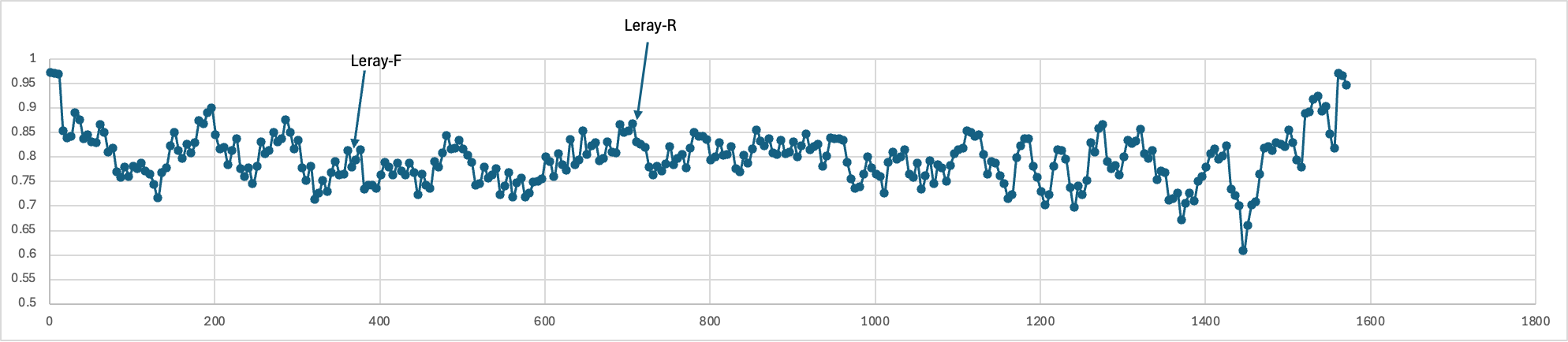


Figure S3. Mean identities along the COXI gene of 441 families of ray-finned fishes.


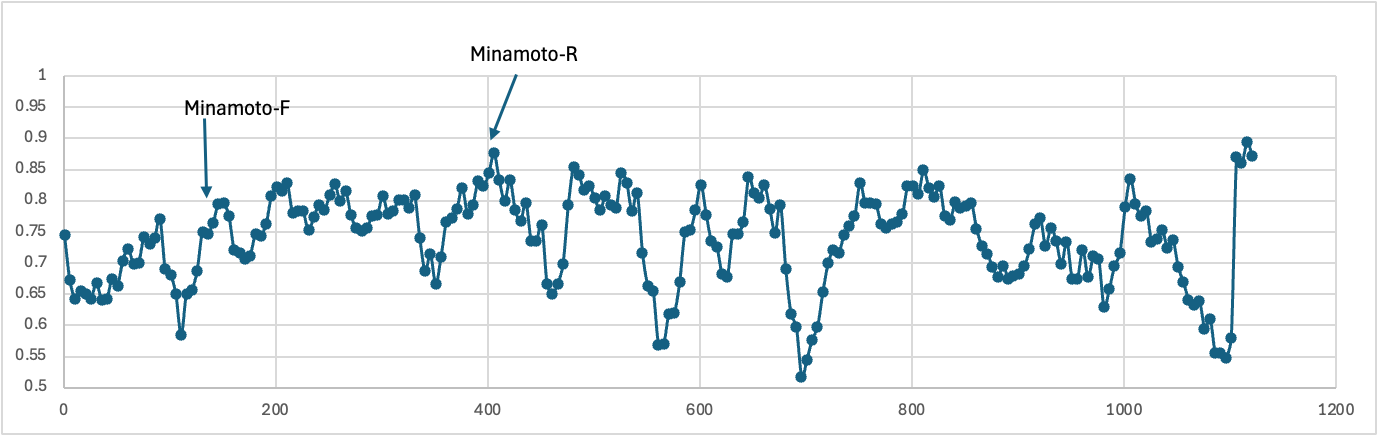


Figure S4. Mean identities along the COXI gene of 466 families of ray-finned fishes.

The raw data are available at Zenodo in the file “Data for Supplementary Text S1.zip” (<https://doi.org/10.5281/zenodo.17911248>).

**References**

Berry TE, Osterrieder SK, Murray DC, Coghlan ML, Richardson AJ, Grealy AK, Stat M, Bejder L, Bunce M. 2017. DNA metabarcoding for diet analysis and biodiversity: A case study using the endangered Australian sea lion (Neophoca cinerea). *Ecol Evol* 7:5435-5453.

Bylemans J, Gleeson DM, Hardy CM, Furlan E. 2018. Toward an ecoregion scale evaluation of eDNA metabarcoding primers: A case study for the freshwater fish biodiversity of the Murray-Darling Basin (Australia). *Ecol Evol* 8:8697-8712.

Evans NT, Olds BP, Renshaw MA, Turner CR, Li Y, Jerde CL, Mahon AR, Pfrender ME, Lamberti GA, Lodge DM. 2016. Quantification of mesocosm fish and amphibian species diversity via environmental DNA metabarcoding. *Mol Ecol Resour* 16:29-41.

Katoh K, Standley DM. 2013. MAFFT multiple sequence alignment software version 7: improvements in performance and usability. *Mol Biol Evol* 30:772-780.

Kelly RP, Port JA, Yamahara KM, Crowder LB. 2014. Using Environmental DNA to Census Marine Fishes in a Large Mesocosm. *PLoS ONE* 9.

Minamoto T, Yamanaka H, Takahara T, Honjo MN, Kawabata Zi. 2012. Surveillance of fish species composition using environmental DNA. *Limnology* 13:193-197.

Miya M, Sato Y, Fukunaga T, Sado T, Poulsen JY, Sato K, Minamoto T, Yamamoto S, Yamanaka H, Araki H, et al. 2015. MiFish, a set of universal PCR primers for metabarcoding environmental DNA from fishes: detection of more than 230 subtropical marine species. *R Soc Open Sci* 2:150088.

Shaw JLA, Clarke LJ, Wedderburn SD, Barnes TC, Weyrich LS, Cooper A. 2016. Comparison of environmental DNA metabarcoding and conventional fish survey methods in a river system. *Biological Conservation* 197:131-138.

Valentini A, Taberlet P, Miaud C, Civade R, Herder J, Thomsen PF, Bellemain E, Besnard A, Coissac E, Boyer F, et al. 2016. Next-generation monitoring of aquatic biodiversity using environmental DNA metabarcoding. *Mol Ecol* 25:929-942.

Vences M, Lyra ML, Perl RGB, Bletz MC, Stanković D, Lopes CM, Jarek M, Bhuju S, Geffers R, Haddad CFB, et al. 2016. Freshwater vertebrate metabarcoding on Illumina platforms using double-indexed primers of the mitochondrial 16S rRNA gene. *Conservation Genetics Resources* 8:323-327.

Wangensteen OS, Palacin C, Guardiola M, Turon X. 2018. DNA metabarcoding of littoral hard-bottom communities: high diversity and database gaps revealed by two molecular markers. *PeerJ* 6:e4705.
