# Supplementary material for: MitoNGS: an online platform to analyze fish metabarcoding data in high resolution
Source: Mol Biol Evol. 2026 Feb 19;43(3):msag046. doi: 10.1093/molbev/msag046 (PMC12961187; doi:10.1093/molbev/msag046)
Supplement: msag046_Supplementary_Data [file msag046_supplementary_data.zip › mitoNGS-20260106 Text S2.docx]

**MitoNGS: an online platform to analyze fish metabarcoding data in high-resolution**

Supplementary Text 2. Results comparison of ASVs and their taxonomy annotations from MitoNGS and DADA2-DECIPHER pipeline

**Methods**

The testing dataset was derived from the Uchidomari River, Okinawa, Japan (Sato, et al. 2018), and comprised two water samples: one filtered using a Millipore Sterivex 0.45 µm filter (accession DRR126154) and the other using a Whatman GF/F 0.70 µm glass fiber filter (accession DRR126155).

DADA2 (v1.30.0) (Callahan, et al. 2016) and DECIPHER (v2.30.0) (Murali, et al. 2018) were installed and executed on the same server as MitoNGS. The scripts and parameter settings for the DADA2–DECIPHER pipeline are provided in the “Data for Supplementary Text S2.zip” file at Zenodo (<https://doi.org/10.5281/zenodo.17911248> ). For MitoNGS, parameters were configured through the web interface as follows:

- Position: [128E, 26N], scope = 10
- minASVabundance = 0%
- minSpeciesIdentity = 99%

Execution time for the DADA2–DECIPHER pipeline was recorded as the “elapsed (wall clock) time” reported by the GNU time command on a Linux system, whereas the runtime of MitoNGS was assessed using the web browser’s developer tools after job submission.

**Results**

45 and 46 ASVs were obtained from MitoNGS and DADA2, respectively, across the two samples. Among them, 28 ASVs shared matched sequences between the two pipelines, accounting for 88.3% and 92.6% of all the reads from the two samples. The detailed statistics of these ASVs was listed in the following table

|  | **MitoNGS** | **DADA2-DECIPHER** |
| --- | --- | --- |
| Running time (seconds) | 4 | 25 |
| all ASVs | 45 | 46 |
| ASVs belonging to fish | 43 | 43 |
| ASVs with matched sequences between MitoNGS and DADA2 | 28 | |
|  | | |
| among the above 28 ASVs | | |
| ASVs resolved to species level | 18 | 11 |
| ASVs resolved to species complexes | 9 | / |
| ASVs resolved to higher taxa | 1 | 17 |

It is obvious that MitoNGS resolved more ASVs to species level than DADA2-DECIPHER. Followings are the results of one-to-one comparison on the 28 ASVs.

**1) 11 ASVs were resolved to species level in both MitoNGS and DADA2-DECIPHER**

All the taxonomy annotations were identical between MitoNGS and DADA2-DECIPHER.

| **MD5 value of ASV sequence** | **Annotation results** | **Abundance**  **(MitoNGS)** |
| --- | --- | --- |
| 007f1bba46f8178b1af382ccf979449a | *Caranx ignobilis* | 51 |
| 0ce97ea4cfdbd76ac08efc1e1196f21f | *Mugil cephalus* | 118 |
| 2978823a11369a51770a778b086c7652 | *Planiliza macrolepis* | 1370 |
| 3c37a2922cd565f1d71196fdb1a845cd | *Kuhlia rupestris* | 746 |
| 901c5752ed71979910e8eeb247c8210d | *Tridentiger kuroiwae* | 3353 |
| 9ec3b8948f811b1372768bd06e9f5d96 | *Mugil cephalus* | 25 |
| a187f0aff70bcd3c378b0e2704a32c6c | *Fibramia amboinensis* | 57 |
| a467a182f6b00d35430448605ac684ba | *Chelonodontops patoca* | 28 |
| aea5608bc3f41fee7d9e9248c7f0df36 | *Scatophagus argus* | 29 |
| c7ad48e69bc4204da31485d50a2659d8 | *Eleotris fusca* | 175 |
| ccba0295c3750a7eafdf573d2d95d64e | *Scatophagus argus* | 125 |

2) **Seven ASVs were resolved to higher taxa in DADA2-DECIPHER while to species level in MitoNGS.**

All the taxonomy annotations were in consistence between MitoNGS and DADA2-DECIPHER. That is, species annotated by MitoNGS belong to the clade annotated by DADA2-DECIPHER.

| **MD5 value of ASV sequence** | **Annotation results** | | **Abundance**  **(MitoNGS)** |
| --- | --- | --- | --- |
|  | **MitoNGS** | **DADA2-DECIPHER** |  |
| 48cbef0a3c5e5147241e59d8560aae0d | *Kuhlia marginata* | *Kuhlia* (genus) | 370 |
| 5c8e65debafc398ae02fd3bfd32fa66b | *Planiliza affinis* | Mugilidae (family) | 183 |
| 94413a0beaa3eea02bf627c5db0136b6 | *Oreochromis niloticus* | *Oreochromis* (genus) | 903 |
| 9d027ca37a4c29705ed1a38d176e0747 | *Rhinogobius similis* | Gobiidae (family) | 682 |
| a05b81375c4359dcbab9414ffe02ee47 | *Rhinogobius sp. MO* | *Rhinogobius* (genus) | 116 |
| 2677f7e4969ba0609c82e966b95d70fe | *Carassius auratus* | *Carassius* (genus) | 148 |
| 8d5a422575e6b5673fb49bd2ebfb38b6 | *Oreochromis niloticus* | *Oreochromis* (genus) | 66 |

Followings were explanations of the differences. The phylogenetic trees were constructed using all the sequences from species or species complexes annotated by MitoNGS and some other species in the same clade (genus or family), using the online MAFFT server (<https://mafft.cbrc.jp/alignment/server/>). Tree clades including the target ASV and annotated species were marked in red, while sequences in heterospecific regions were marked in purple.

a) results filtered by hits in heterospecific regions (the first five ASVs)

ASV 48cbef0a3c5e5147241e59d8560aae0d

The only alternative top hit from other species with equal bit-score was MZ598435 (from *Kuhlia malo*). The target region of other *Kuhlia* species were in high divergence. Therefore, annotating as genus *Kuhlia* in DECIPHER was ambiguous. There were no other available 12S rRNA sequences from *Kuhlia malo*. However, *Kuhlia malo* is endemic to French Polynesia, far away from Japan. The reference sequence MZ598435 has no related publication literatures. Therefore, it is reasonable to suspect that MZ598435 is misannotated, and this ASV should be annotated as *Kuhlia marginata*.

ASV 5c8e65debafc398ae02fd3bfd32fa66b

The only alternative top hit from other species with equal bit-score was MG587040 (*Chelon carinatus*). In FishBase, it is recorded as *Planiliza carinata* (<https://www.fishbase.org/summary/SpeciesSummary.php?ID=13673>), indicating close relationship of the genus *Chelon* and *Planiliza*. against other clades in the Mugilidae family. Therefore, annotating as the Mugilidae family in DECIPHER was ambiguous. There were no other available 12S rRNA sequences from *Chelon carinatus*. However, *Chelon carinatus* is endemic to the red sea, far away from Japan. The reference sequence MG587040 has no related publication literatures, and its source region is unknown. Therefore, it is reasonable to suspect that MG587040 is misannotated, and this ASV should be annotated as *Planiliza affinis*.

ASV 94413a0beaa3eea02bf627c5db0136b6

The only alternative top hit from other species with equal bit-score was MZ598440 (*Oreochromis mossambicus*). Both *Oreochromis niloticus* and *O. mossambicus* are distributed worldwide, and the specimen of MZ598440 was originated from French Polynesia. The phylogenetic relationship of different *Oreochromis* species were mixed. This ASV was in closest relationship with the Japan ecotype of *Oreochromis niloticus*. The reference sequence MZ598440 might be misannotated, but the possibility that it originated from a native ecotype of *O. mossambicus* could not be completely excluded. Further classification needs to wait for more completed reference sequences.

ASV 9d027ca37a4c29705ed1a38d176e0747

The only alternative top hit from other species with equal bit-score was MT210646 (*Acanthogobius flavimanus*). The origin region and related publication of MT210646 were unclear. It was in high divergence with other sequences from *A. flavimanus*. Therefore, it is reasonable to suspect that MT210646 is misannotated, and this ASV should be annotated as *Rhinogobius similis* rather than Gobiidae, the lowest common ancestor (LCA) of *Acanthogobius flavimanus* and *Rhinogobius similis*.

ASV a05b81375c4359dcbab9414ffe02ee47

The only alternative top hit from other species with equal bit-score was LC601884 (*Rhinogobius* sp. BB). There were no other available 12S rRNA sequences from *Rhinogobius* sp. BB. Since *Rhinogobius* sp. MO was also an unclassified species, annotation as *Rhinogobius* (genus) was also acceptable.

b) results filtered by hits in non-binomial species (the last two ASVs)

| **MD5 value of ASV sequence** | **Species from top hits** |
| --- | --- |
|  |  |
| 2677f7e4969ba0609c82e966b95d70fe | *Carassius auratus* (reported in MitoNGS)  *Carassius* sp. 'Ginbuna' (LC049911) (excluded)  *Carassius* sp. sensu Hosoya, 2013 (LC765847) (excluded) |
| 8d5a422575e6b5673fb49bd2ebfb38b6 | *Oreochromis niloticus* (reported in MitoNGS)  *Oreochromis* sp. KM-2006 (AP009126) (excluded)  *Oreochromis* sp. 'red tilapia'(GU477631) (excluded)  *Oreochromis* sp. OCNFW28 (MZ605473) (excluded) |

3) **Nine ASVs were resolved to higher taxa in DADA2-DECIPHER while to species complexes in MitoNGS.**

All the taxonomy annotations were in consistence between MitoNGS and DADA2-DECIPHER. That is, species complexes annotated by MitoNGS belong to the clade annotated by DADA2-DECIPHER.

| **MD5 value of ASV sequence** | **Annotation results** | | **Abundance**  **(MitoNGS)** |
| --- | --- | --- | --- |
|  | **MitoNGS** | **DADA2-DECIPHER** |  |
| 297d0fb97f0d8b00a20e8438ced54f61 | *Carassius auratus*  *Cyprinus carpio*  *Carassius gibelio*  *Carassius carassius* | Cypriniformes (order) | 256 |
| 3d041325e75a2e343d0a36998078cca8 | *Pterygoplichthys pardalis*  *Pterygoplichthys disjunctivus* | *Pterygoplichthys* (genus) | 110 |
| 416dddee5acae6e786ed521f406a7ff1 | *Cyprinus carpio*  *Pseudorasbora parva*  *Cyprinus acutidorsalis* | Cypriniformes (order) | 1445 |
| 5edfa0d3c088cea1c99b8e5683b40836 | *Caranx melampygus*  *Caranx tille*  *Caranx sexfasciatus* | *Caranx* (genus) | 47 |
| 72fbe6a842ce09d21b81e804b816ed2c | *Rhinogobius yonezawai*  *Rhinogobius brunneus*  *Rhinogobius yaima* | *Rhinogobius* (genus) | 158 |
| 9ef8f481dee3c044590a1ccb265b1dcd | *Anguilla interioris*  *Anguilla marmorata* | *Anguilla* (genus) | 157 |
| cdb8a7cb3cc17aa9d65119bbb07673b0 | *Anguilla interioris*  *Anguilla marmorata* | *Anguilla* (genus) | 1116 |
| d0c2209fda13475ec7842313a14e3b7c | *Luciogobius guttatus*  *Luciogobius ryukyuensis* | *Luciogobius* (genus) | 121 |
| e14784f1f3b0021d6de15bde7bd4f90f | *Acanthopagrus sivicolus*  *Acanthopagrus schlegelii* | *Acanthopagrus* (genus) | 95 |

Followings were explanations of the differences:

ASV 297d0fb97f0d8b00a20e8438ced54f61

This ASV had multiple top hits with equal bit-score from four species of the Cyprinidae family: *Carassius auratus*, *Cyprinus carpio*, *Carassius gibelio* and *Carassius carassius*. There were also top hits from inter-genus hybrids such as *Carassius auratus* x *Cyprinus carpio* (GU186888), with no family rank set in the NCBI Taxonomy Database. Therefore, the LCA of all the top hits led to the Cypriniformes order in DECIPHER’s result. Since the Cypriniformes order includes more than 7,000 species, the result of MitoNGS is significantly clearer.

ASV 3d041325e75a2e343d0a36998078cca8

This ASV had top hits with equal bit-score from three species of the *Pterygoplichthys* genus: *P. pardalis*, *P. disjunctivus* and *P. anisitsi*. Reference sequences of *P. anisitsi* were from a single study (KT239003, KT239004, and KT239005) (Parente, et al. 2017), therefore not included in the result of MitoNGS since they might be heterospecific. Other sequences from *Pterygoplichthys* genus were in highly divergence, Therefore, it is reasonable to annotate as *P. pardalis* / *P. disjunctivus* instead of the whole *Pterygoplichthys* genus, which includes more than 70 species.

ASV 416dddee5acae6e786ed521f406a7ff1

This ASV had multiple top hits with equal bit-score from two species of the Cyprinidae family: *Cyprinus carpio* and *Cyprinus acutidorsalis,* and one Gobionidae species: *Pseudorasbora parva*. The LCA of them led to the Cypriniformes order in DECIPHER’s result. Since the Cypriniformes order includes more than 7,000 species, the result of MitoNGS is significantly clearer.

ASV 5edfa0d3c088cea1c99b8e5683b40836

This ASV had multiple top hits with equal bit-score from four species of the *Caranx* genus: *C. melampygus*, *C. tille*, *C. sexfasciatus* and *C. caninus* (OQ846170). There were no other available 12S rRNA sequences from *C. caninus*, therefore not included in the result of MitoNGS since it might be heterospecific. Other sequences from *Caranx* genus were in highly divergence. Therefore, it is reasonable to annotate as *C. melampygus* / *C. tille* / *C. sexfasciatus* instead of the whole *Caranx* genus, which includes more than 30 species.

ASV 72fbe6a842ce09d21b81e804b816ed2c

This ASV had multiple top hits with equal bit-score from three species of the *Rhinogobius* genus: *R. yonezawai*, *R. brunneus* and *R. yaima*. They formed a monophyletic clade. Other sequences from *Rhinogobius* genus were in highly divergence. Therefore, it is reasonable to annotate as *R. yonezawai* / *R. brunneus* / *R. yaima* instead of the whole *Rhinogobius* genus, which includes more than 100 species.

ASV 9ef8f481dee3c044590a1ccb265b1dcd and cdb8a7cb3cc17aa9d65119bbb07673b0

These two ASVs had multiple top hits with equal bit-score from two species of the *Anguilla* genus: *A. interioris* and *A. marmorata*. They formed a monophyletic clade. Other sequences from *Anguilla* genus were in highly divergence. Therefore, it is reasonable to annotate as *A. interioris* / *A. marmorata* instead of the whole *Anguilla* genus, which includes more than 20 species.

ASV d0c2209fda13475ec7842313a14e3b7c

This ASV had multiple top hits with equal bit-score from two species of the *Luciogobius* genus: *L. guttatus* and *L. ryukyuensis*. They formed a monophyletic clade. Other sequences from *Luciogobius* genus were in highly divergence. Therefore, it is reasonable to annotate as *L. guttatus* / *L. ryukyuensis* instead of the whole *Luciogobius* genus, which includes more than 30 species.

ASV e14784f1f3b0021d6de15bde7bd4f90f

This ASV had multiple top hits with equal bit-score from two species of the *Acanthopagrus* genus: *A. sivicolus* and *A. schlegelii*. They formed a monophyletic clade. Other sequences from *Acanthopagrus* genus were in highly divergence. Therefore, it is reasonable to annotate as *A. sivicolus* / *A. schlegelii* instead of the whole *Acanthopagrus* genus, which includes more than 20 species.

**4) One ASV were resolved to higher taxa in both DADA2-DECIPHER and MitoNGS**

The top hit of ASV ea389a8b7e26012b099b6a1a7ee7282c was from the inter-genus hybrid specimen Carassius auratus x Cyprinus carpio (AY694420). The similarity was 98.8%, lower than the default 99% threshold to be resolved to species-level. Therefore, in MitoNGS it was reported in the higher-taxa section, while in DECIPHER it was reported as “unclassified Cypriniformes”.

**References**

Callahan BJ, McMurdie PJ, Rosen MJ, Han AW, Johnson AJ, Holmes SP. 2016. DADA2: High-resolution sample inference from Illumina amplicon data. *Nat Methods* 13:581-583.

Murali A, Bhargava A, Wright ES. 2018. IDTAXA: a novel approach for accurate taxonomic classification of microbiome sequences. *Microbiome* 6:140.

Parente TE, Moreira DA, Magalhães MGP, de Andrade PCC, Furtado C, Haas BJ, Stegeman JJ, Hahn ME. 2017. The liver transcriptome of suckermouth armoured catfish (Pterygoplichthys anisitsi, Loricariidae): Identification of expansions in defensome gene families. *Marine Pollution Bulletin* 115:352-361.

Sato Y, Miya M, Fukunaga T, Sado T, Iwasaki W, Kumar S. 2018. MitoFish and MiFish Pipeline: A Mitochondrial Genome Database of Fish with an Analysis Pipeline for Environmental DNA Metabarcoding. *Molecular Biology and Evolution* 35:1553-1555.
